# Expression Atlas update—a database of gene and transcript expression from microarray- and sequencing-based functional genomics experiments

**DOI:** 10.1093/nar/gkt1270

**Published:** 2013-12-04

**Authors:** Robert Petryszak, Tony Burdett, Benedetto Fiorelli, Nuno A. Fonseca, Mar Gonzalez-Porta, Emma Hastings, Wolfgang Huber, Simon Jupp, Maria Keays, Nataliya Kryvych, Julie McMurry, John C. Marioni, James Malone, Karine Megy, Gabriella Rustici, Amy Y. Tang, Jan Taubert, Eleanor Williams, Oliver Mannion, Helen E. Parkinson, Alvis Brazma

**Affiliations:** European Molecular Biology Laboratory, European Bioinformatics Institute, EMBL-EBI, Hinxton, CB10 1SD, UK

## Abstract

Expression Atlas (http://www.ebi.ac.uk/gxa) is a value-added database providing information about gene, protein and splice variant expression in different cell types, organism parts, developmental stages, diseases and other biological and experimental conditions. The database consists of selected high-quality microarray and RNA-sequencing experiments from ArrayExpress that have been manually curated, annotated with Experimental Factor Ontology terms and processed using standardized microarray and RNA-sequencing analysis methods. The new version of Expression Atlas introduces the concept of ‘baseline’ expression, i.e. gene and splice variant abundance levels in healthy or untreated conditions, such as tissues or cell types. Differential gene expression data benefit from an in-depth curation of experimental intent, resulting in biologically meaningful ‘contrasts’, i.e. instances of differential pairwise comparisons between two sets of biological replicates. Other novel aspects of Expression Atlas are its strict quality control of raw experimental data, up-to-date RNA-sequencing analysis methods, expression data at the level of gene sets, as well as genes and a more powerful search interface designed to maximize the biological value provided to the user.

## INTRODUCTION

Expression Atlas is a further development of our previous version of Gene Expression Atlas (1), launched by the European Bioinformatics Institute (EBI) in 2008, and continues its original remit as a value-added database for querying differential gene expression across tissues, cell types and cell lines under various biological conditions. These include developmental stages, physiological states, phenotypes and diseases and cover multiple organisms. Expression Atlas is developed with a view to accommodate data from multi-omics experiments, such as proteomics. High-quality microarray and RNA-sequencing data in Expression Atlas continues to come from ArrayExpress (2), including data imported from GEO (3). Differential expression is reported for both coding and non-coding transcripts. The sample attributes and experimental factors (i.e. conditions under study) are systematized and mapped to the Experimental Factor Ontology [EFO (4)].

In particular, Expression Atlas introduces the concept of baseline expression—the abundance of each gene and splice variant in healthy or untreated tissues, cell types or cellular components. Baseline expression is reported within a species-specific context of selected large RNA-sequencing experiments and provides a useful reference for the user when considering differential expression data.

Expression Atlas continues to analyse and report statistically robust differential expression for both coding and non-coding transcripts. However, the biological relevance of these data has been vastly improved due to an in-depth manual curation of the experimental intent that for each differential experiment yields a set of ‘contrasts’, i.e. instances of differential pairwise comparisons between two sets of biological replicates—the ‘reference’ (e.g. ‘healthy’ or ‘wild type’) set and a ‘test’ set (e.g. ‘diseased’ or ‘mutant’). Each of these sets is typically described by a number of sample attributes and experimental factors. For example, all biological replicates treated with a test compound may be compared with untreated samples. Statistical analysis is then performed, providing *P*-values and, (for microarray only) *t-*statistics, linking each gene to differential contrasts in each experiment.

Another novel aspect of Expression Atlas is its focus on quality control of raw experimental data and of experimental design. A minimum acceptable number of biological sample replicates (three) is also enforced to ensure sufficient statistical power to detect differential expression. Before submission into analysis pipelines, all experimental raw data undergo quality control. In the case of RNA-sequencing experiments, poor quality reads and those originating from contamination are excluded from further analysis. Outlier arrays in microarray experiments are also removed before manual contrast identification and statistical analysis.

The focus on the quality of raw data and experimental design has led to exclusion of low-quality experiments. The manual curation of contrasts for all eligible experiments is also on-going, leading to a temporary reduction in the number of experiments in Expression Atlas.

Support of reproducible analysis is provided for each experiment by listing analysis methods and versions used for processing its raw data, offering links to source code where possible, as well as showing the version of Ensembl genome reference used for mapping (for RNA-sequencing experiments), and the version of miRBase (5) release from which probe-set to microRNA mappings was taken for microRNA microarray experiments. The user should thus be able to reproduce the results presented in Expression Atlas, by analysing the raw experimental data using the methods listed for that experiment.

Expression Atlas search interface allows for querying gene, splice variant or protein attributes (including organism), at the level of individual genes or whole gene sets. The user can also search for sample attributes and experimental factors. Both baseline and differential components of Expression Atlas are queried by default. The experiments returned are those in which the queried sample attributes match either the studied healthy or untreated biological conditions, e.g. tissues or cell types (baseline expression), or match either a ‘test’ or a ‘reference’ side of a differential contrast (differential expression). Finally, the set of queried experiments can be restricted by providing a list of accessions, keywords or the species of samples studied in them.

**Figure 1.**
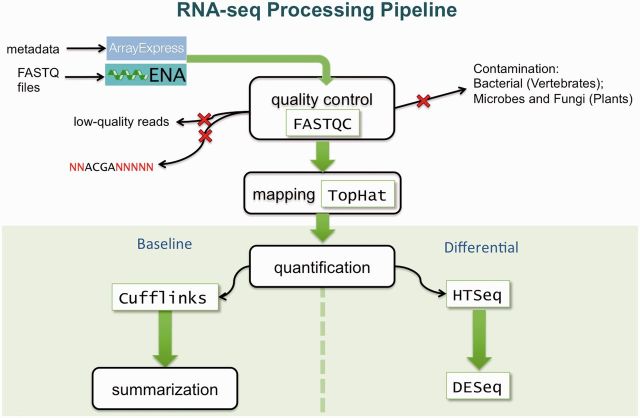
The RNA-seq processing pipeline used to generate data for Expression Atlas. The experimental metadata is retrieved from ArrayExpress. The raw FASTQ files, retrieved from European Nucleotide Archive, undergo a quality control procedure via FASTQC package to remove low-quality reads and uncalled bases. Subsequently, contaminated reads (e.g. bacterial in the cases of vertebrate samples) are removed. TopHat 1 is used for mapping the reads to the reference genome, Cufflinks 1 quantifies baseline expression for genes and transcripts and HTseq quantifies expression used for subsequent differential expression analysis with DESeq. The final (summarized) baseline expression count for a gene in a condition is a median across first technical replicates, then across biological replicates corresponding to that condition.

The RNA-seq processing pipeline used to generate data for Expression Atlas is shown in Figure 1. The full details of material and methods used to generate expression data shown in Expression Atlas interface are available in the Supplementary Material.

## RESULTS

### Data

As of 24 September 13, Expression Atlas contains highly curated data from 214 experiments, including four baseline RNA-sequencing experiments (nine species) and 210 differential experiments (13 species). Baseline experiments include *Illumina Body Map* (http://www.ebi.ac.uk/gxa/experiments/E-MTAB-513) and *Encode Cell Lines* (http://www.ebi.ac.uk/gxa/experiments/E-GEOD-26284). Differential experiments include 10 RNA-sequencing and 200 microarray experiments—mainly single-channel experiments performed on gene arrays. Finally, microarray experiments studying microRNAs are also available (e.g. http://www.ebi.ac.uk/gxa/experiments/E-TABM-713).

### New user interface features

Expression Atlas offers a separate page for each experiment, as well as pages presenting baseline and differential expression data for each gene, protein, gene set (e.g. REACTOME pathway) and experimental condition (e.g. ‘heart’) stored in Atlas.

#### Baseline expression (Figure 2)

**Figure 2.**
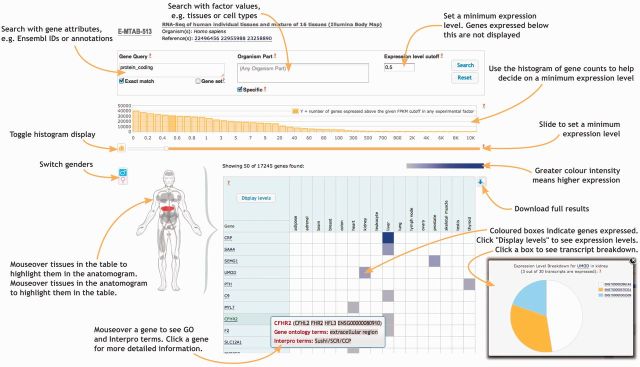
Example baseline expression experiment page, with help annotations—*Illumina Body Map.* (For further information see: http://www.ebi.ac.uk/gxa/help/baseline-atlas.html).

Users can search a baseline experiment with gene names, protein accessions, gene, protein or splice variant identifiers, keywords, biotypes (e.g. ‘protein coding’), GO and InterPro terms as well as Reactome pathway IDs. Optionally, each term (e.g. REACTOME pathway ID) can be interpreted as a gene set, offering the user an aggregated expression level across all genes in each queried gene set. Users may also search using studied experimental conditions (e.g. tissue in Figure 2). By default, search results are ordered such that genes that are most specifically expressed in the experimental condition(s) of interest are at the top. This is implemented by rewarding higher expression in the conditions of interest and as low as possible expression in the remaining conditions. Optionally, the user may wish to search for ‘non-specific’ expression—in this scenario genes with high expression in query conditions are not only rewarded but also not penalized for high expression in non-query conditions. This type of query typically returns ‘house-keeping’ genes at the top of the results table, i.e. those with high levels of expression in the majority of experimental conditions. Expression levels below the displayed FPKM cut-off (0.5 by default) are treated as background (i.e. ‘noise’). The user is free to select a different expression level cut-off—a histogram breaking down the number of genes expressed above a given cut-off is included to help the user decide which cut-off to use for their query of interest. As FPKMs are already a gross approximation of gene expression, the resulting matrix encodes the expression level by way of a heatmap, though the actual FPKM values can be displayed and are downloadable from the experiment page. Finally, clicking on a non-empty heatmap cell shows a breakdown of the three most abundant splice variants for the corresponding gene and experimental condition.

#### Differential expression (Figure 3)

Users may search a differential experiment by the same gene properties and keywords as listed earlier in text for baseline experiments, additionally selecting the type of differential expression of interest (up/down by default). A differential contrast dropdown is also available—the default search is for differential expression in any contrast, but the user can also choose one or more contrast of interest. By default, the search returns first genes that are differentially expressed most specifically in the queried contrast(s). This is achieved by promoting to the top genes with lowest *P-values* in the contrast(s) of interest and at the same time penalizing genes with low *P*-values in the remaining contrasts. Optionally, the user may perform a ‘non-specific’ search, in which genes with lowest *P-values* in the selected contrast(s) come first, irrespective of whether they are reported with low *P*-values in the remaining contrasts. The results of this analysis are presented to the user in a matrix, with genes (and design elements—for microarray only) as row labels, and contrasts as column labels. The results are sorted by *P*-value; the *t*-statistics and log_2_-fold changes are also available. As part of the differential analysis, ‘MA’ plots are shown for the default FDR of 0.05. The user is able to choose a different FDR and observe in the resulting matrix, what effect this has had on the results. The differential experiment page offers downloads of analytics data as well as raw counts (RNA-sequencing), normalized expression values (one-colour microarray) and log_2_-ratios (two-colour microarray), respectively. Finally, experimental conditions for each contrast can be viewed via mouse-over on contrast column headers, in the results matrix, and on the experiment design page, available via a button in the top-right corner of the experiment page.

**Figure 3.**
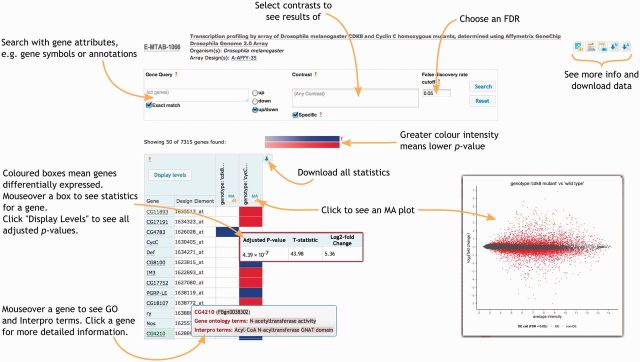
Example differential expression page, with help annotations*—Transcription profiling by array of Drosophila melanogaster CDK8 and Cyclin C* homozygous mutants, determined using ‘Affymetrix GeneChip Drosophila Genome 2.0 Array’. (For further information see: http://www.ebi.ac.uk/gxa/help/differential-atlas.html). Genes that were called as differentially expressed at FDR < 0.05 are shown in red in the MA plot.

#### Gene/protein/gene-set page

**Figure 4.**
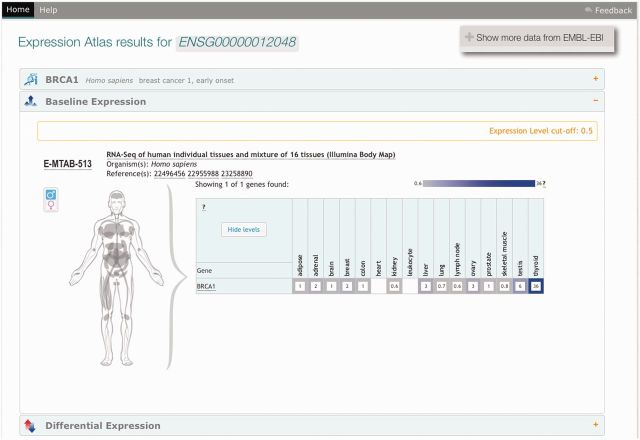
Baseline expression on summary page example for human *BRCA1* gene: http://www.ebi.ac.uk/gxa/genes/ENSG00000012048.

**Figure 5.**
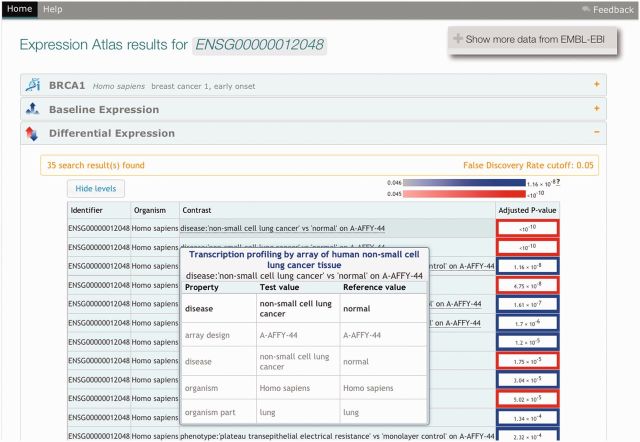
Differential expression on summary page for human *BRCA1* gene: http://www.ebi.ac.uk/gxa/genes/ENSG00000012048.

For each gene, protein and gene set (e.g. Reactome pathway ID), Expression Atlas provides a summary page that contains, at most, three separate panes (Figure 4). The top pane contains extensive annotation for the represented bio-entity, including links to external resources, its orthologues and so forth. The middle pane shows baseline expression information from the representative baseline experiment in which the bio-entity was studied. For gene sets, the aggregated expression levels across all genes in the set are shown for each experimental condition. Finally, the bottom pane (Figure 5) shows differential expression, sorted by *P*-value, across all contrasts in experiments available in Expression Atlas. Mouse-over on a contrast description shows experimental conditions describing the test and the reference sides of that contrast (shown in Figure 5 for the top contrast); clicking on a contrast takes the user to the page of the experiment from which the analytics were retrieved.

#### Experiment list page

This page (http://www.ebi.ac.uk/gxa/experiments) presents a sortable and searchable list of all experiments currently loaded in Expression Atlas, documenting, among other things, experiment type (baseline or differential), the number of assays analysed for that experiment, the organisms and experimental conditions studied, the number of contrasts identified (differential experiments only) and the array designs used in the experiment (microarray only).

### Atlas infrastructure developments

#### Software availability

The Expression Atlas software is designed to run in-house only. However, the software source code can be accessed via http://github.com/gxa/atlas.

#### Release process

Gene Expression Atlas, described in our previous update, released its data and software on a monthly basis. Expression Atlas will also release data regularly, providing individual experimental data and tar-gzip snapshots of all the data (for ease of download) on the EMBL-EBI FTP server (ftp://ftp.ebi.ac.uk/pub/databases/microarray/data/atlas). The web services software will be released regularly, with appropriate release notes notifying users of functionality changes.

## FUTURE DIRECTIONS

### Protein expression

Expression Atlas is intended as a multiomics, and in particular as a functional genomics and proteomics, resource, incorporating expression of not only genes but also splice variants and proteins. Although the quantitation and statistical analysis of gene expression methods is relatively mature and well established, the equivalent methods for protein detection, quantification and statistical analysis are still active areas of research. Consequently, in the first instance, we will include protein expression data as additional information to the transcriptomics data in the baseline component of Expression Atlas only. EFO will be used to identify data sets with corresponding sample descriptions in PRIDE database (6). Expression of each protein in those sets will be shown within the context of the baseline expression of the particular gene coding for the protein in the corresponding experimental condition. Appropriate provenance will be attributed to each source of protein expression data within Expression Atlas interface.

### Baseline expression data improvements

We plan to increase our baseline expression coverage to experiments in novel species, containing greater resolution of studied factors, e.g. tissues, as well as with greater biological replication of studied samples—in aid of more robust analysis results presented to the user. The baseline expression analysis will also include data sets that study heterogeneity among individuals and, for example, tissues, focusing on variation data, expression quantitative trait loci and mutations.

### Expression visualization improvements

We will make transcript expression levels more prominent in our experiment pages, focusing on genome browser coverage views of expression—allowing the user to observe in detail how expression is distributed across different exons and transcripts of a given gene.

### Baseline expression aggregation

We are working on methods to aggregate expressions of a gene, in a given experimental condition, across all applicable RNA-sequencing experiments, so that a single expression level for that ‘gene-experimental condition’ combination can be shown to the user.

### Gene set enrichment analysis

Currently, only baseline expression summaries for gene sets are offered in Expression Atlas interface. Pre-computed gene set enrichment analysis results in the context of differential expression will be offered, for example, InterPro, GO terms and REACTOME pathways. The results of this analysis will be shown in the corresponding gene set summary page. Users will also be able to submit an arbitrary set of genes to quantify enrichment against all contrasts/differential gene sets present in Expression Atlas. Such queries may be submitted together with experimental conditions to restrict the set of contrasts to analyse the enrichment in.

### Hom(e)ologue expression

The Expression Atlas interface will facilitate gene co-expression analysis, including paralogues and homeologues where applicable, as well as comparative analysis of expression of orthologues.

### MicroRNA RNA-sequencing experiments

The pipeline used to process RNA-sequencing data for Expression Atlas will be enhanced to analyse microRNA RNA-sequencing experiments. Subsequently, good quality microRNA RNA-sequencing experiments available in ArrayExpress will be re-processed and included in Expression Atlas.

## SUPPLEMENTARY DATA

Supplementary Data are available at NAR Online.

## FUNDING

European Molecular Biology Laboratory (EMBL) member states; the National Science Foundation [Gramene, 1127112]; the European Community's FP7 EurocanPlatform [260791]; and by GEUVADIS [261123]. Funding for open access charge: EMBL central publication budget.

*Conflict of interest statement*. None declared.
